# Antimicrobial peptides for tuberculosis in a post-antibiotic era: from in vitro promise to therapeutic potential

**DOI:** 10.1038/s44259-026-00238-z

**Published:** 2026-08-18

**Authors:** Gama Dominic, Yasmin Neves Vieira Sabino, Mustasim Famous, Omololu Ebenezer Fagunwa, Rachel M. Wheatley, Ralf Bauer, Suzanne Hingley-Wilson, Linda B. Oyama

**Affiliations:** 1 Institute for Global Food Security, School of Biological Sciences, Queen’s University Belfast, Belfast, UK; 2 University of Strathclyde, Technology & Innovation Centre, Glasgow, UK; 3 School of Biosciences, University of Surrey, Guildford, Surrey, UK; 4 Department of Animal Science, Khulna Agricultural University, Khulna, Bangladesh

**Keywords:** Computational biology and bioinformatics, Diseases, Drug discovery, Microbiology

## Abstract

Tuberculosis remains the leading cause of death from a single infectious pathogen, driven by prolonged treatment regimens and escalating drug resistance. Antimicrobial peptides represent a promising alternative therapeutic class with diverse mechanisms and limited resistance emergence. This review distinguishes anti-mycobacterial from anti-*Mycobacterium tuberculosis* peptides and systematically analyses 22 candidates curated in the APD6 alongside additional literature reports. We evaluate their structural and physicochemical determinants of activity, mechanistic evidence in vitro and in vivo, and synergy with conventional agents. Finally, we discuss rational design strategies, machine learning-guided optimisation, and translational barriers that must be overcome to realise peptide-based tuberculosis therapeutics.

**Figure:**
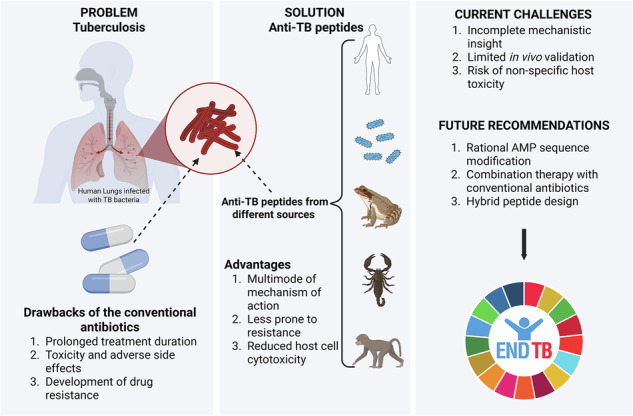

## Tuberculosis: a global health concern

Tuberculosis (TB), caused mainly by *Mycobacterium tuberculosis* (Mtb), remains the deadliest infectious disease of the 21st century^1^. Around one quarter of the global population is estimated to be infected with Mtb^2^. According to statistics from the World Health Organization (WHO), 10.7 million people acquired new Mtb infections in 2024^1^. Additionally, TB caused ~1.23 million deaths across the world in 2024, being the leading cause of death from a single infectious agent^1^.

Mtb is a facultative intracellular bacterium, which can live and proliferate inside phagocytes like macrophages, destabilising the functional properties of these essential innate immune cells^3^. Mtb can survive inside the host in its inactive form (latent TB infection)^4^, and whenever the host’s immunity is disturbed, it can take up its active form, driving symptoms such as a prolonged cough, chest pain, fever, weight loss, haemoptysis, fatigue, night sweat and loss of appetite^5–7^. Although Mtb is the primary pathogen responsible for human TB, the disease can also be caused by phylogenetically related bacilli that belong to a larger group called the *Mycobacterium tuberculosis* complex (MTBC). This complex includes *Mycobacterium bovis, Mycobacterium africanum, Mycobacterium canettii, Mycobacterium microti, Mycobacterium caprae, Mycobacterium pinnipedii, Mycobacterium orygis and Mycobacterium mungi*^8,9^.

**Fig. 1:**
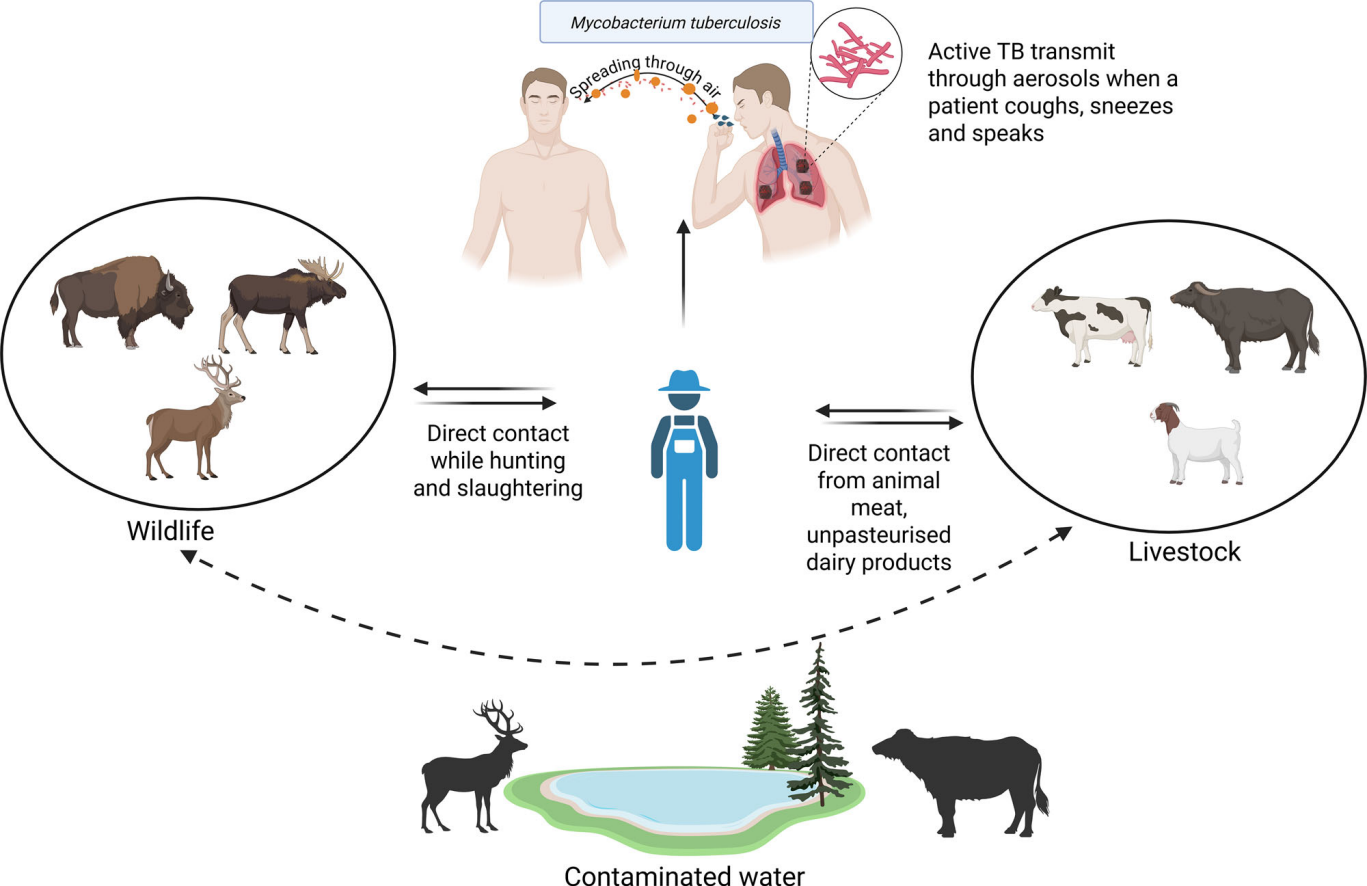
One Health challenge associated with TB. TB is an airborne disease primarily caused by *Mycobacterium tuberculosis*, which is human-adapted and has no animal reservoir. However, other members of *Mycobacterium tuberculosis* complex MTBC such as *Mycobacterium bovis*, *Mycobacterium africanum* can also affect humans through close contact with livestock or consumption of unpasteurised animal products. The complex interphase between humans, animals (both livestock and wildlife), and the environment makes TB a One Health challenge. This image was created using BioRender.

Tuberculosis does not only affect humans, but also animals, for example, with infections caused by *Mycobacterium bovis* (Bovine-TB)^10^ and *Mycobacterium avium* subsp. *paratuberculosis* (Johne’s disease)^11^. Bovine-TB has been reported in the agri-food industry globally, including in Great Britian^12^, the Republic of Ireland^12^, New Zealand^13^, India^14^, Australia^15^ and several parts of Africa^16–19^, while it is endemic in Cameroon^20^. Whereas Johne’s disease is more prevalent in the US^21^, Canada^22^, European Countries^23^, Australia^24^, South Africa^25^ and India^26^. Airborne transmission of TB can occur, mainly from close interaction of humans with animals, and by the consumption of non-pasteurised milk and milk products, hence making TB a One Health challenge (Fig. 1). This underscores the WHO’s End TB Strategy, which aims for a 95% reduction of TB deaths and 90% drop in new TB incidences by 2035.

## Current challenges in tuberculosis prevention and treatment

Many drug-sensitive Mtb infections are curable if diagnosed and treated on time^27^. Vaccination is likely one of the most cost-effective methods for curtailing this global health threat by decreasing TB incidence. The Bacillus Calmette Guérin (BCG) vaccine is currently the only available TB vaccine, and it provides partial and variable protection against human TB^28^, showing efficacy only for younger children against TB meningitis, disseminated TB and leprosy in regions where TB is endemic^29,30^. However, its efficacy in adults is highly variable^31,32^. Moreover, a randomised controlled trial in Brazil showed that revaccination with BCG in puberty in a population vaccinated with BCG during birth does not enhance protection^33^. Currently, 18 novel TB vaccines are at different phases of clinical trials to try and tackle this challenge^1^.

The discovery of streptomycin in 1943 marked the beginning of TB drug discovery^34^. Later in 1952, the first combination treatment (triple therapy, including streptomycin, para-aminosalicylic acid and isoniazid (INZ) was proposed^35,36^. In the 1960s, ethambutol (EMB) replaced para-aminosalicylic acid in the therapeutic scheme for TB as it proved to be more effective and better tolerated^37^. By the 1970s, the inclusion of rifampicin (RIF) in TB treatment reduced the treatment duration from 12-18 months to nine months^38,39^. This duration was further shortened to six months with the later addition of pyrazinamide (PZA) to the first-line regimen^40,41^.

Given the ineffectiveness of current prevention measures, the availability of effective drugs to treat TB is even more important. Since 2022, the WHO has recommended the use of two shortened treatment regimens for rifampicin-susceptible TB. These include the combination of INZ, rifapentine (RPT), moxifloxacin, and PZA for the general treatment of TB, and a 4-month regimen containing INZ, RIF, PZA and EMB for children and adolescents (3–16 years) with non-severe TB^2^. Although this combination therapy is designed to effectively eliminate the TB bacteria, drug resistance continues to rise. The rise of antibiotic-resistant TB strains has prompted the need for second-line treatment strategies.

Mtb strains resistant to the first-line drugs INH and RIF are categorised as multidrug-resistant TB (MDR-TB), whereas rifampicin-resistant (RR-TB) refers to the TB caused by strains resistant to RIF alone^42^. People suffering from drug-resistant TB infections need to be treated with regimens containing second-line drugs, such as bedaquiline (BDQ) and fluoroquinolones (FQs). Since 2018, the WHO has recommended all-oral regimens for the treatment of MDR-TB/RR-TB. For patients with or without resistance to FQs, the 6-month all-oral regimen consists of BQD, pretomanid, linezolid and moxifloxacin (BPaLM regimen). For patients with confirmed FQs resistance, moxifloxacin is eliminated, and the treatment is continued with BPaL^2,42^. Moreover, for patients whose TB strains are susceptible to FQs, a 9-month all-oral regimen is recommended. This includes BDQ for the first six months, in combination with levofloxacin or moxifloxacin, ethionamide, EMB, high-dose INZ, PZA, and clofazimine, typically administered for four months but may be extended to six months if the patient remains sputum smear-positive at the end of the fourth month^2,42^. Additionally, Mtb strains resistant to RIF, any FQs and at least one of BDQ or linezolid are defined as extensively drug-resistant TB (XDR-TB)^2^. Longer individualised regimens with treatment durations of at least 18 months are recommended when a patient shows intolerance to the 6-month, 9-month and key components of the aforementioned regimens. Individualised medicines are designed based on a structured treatment hierarchy of second-line TB regimens, drug resistance profile and the patient’s previous health records^42^. Based on the Nix-TB trial^43^, BPaL was approved by the US FDA (Food and Drug Administration) for patients with XDR-TB, which is recommended to be administered only when BDQ and linezolid have not been used previously as a substitute to the longer regimen^44^. However, many of these second-line drugs exhibit increased toxicity and are more expensive than the first line drugs^45^, warranting the development of synergistic treatments that can reduce the dosage given and treatment time.

Another challenge in the advancement of TB treatment is that TB research and the development of effective candidates require Biosafety Level III laboratories. This is further hampered by the slow-growing nature of the tubercle bacterium. Consequently, the use of proxy organisms for Mtb research, e.g., *Mycobacterium smegmatis (M. smegmatis*), is common. This has implications for TB drug development as it could lead to the loss of potent anti-TB drug discovery. For instance, certain compounds, including antimicrobial peptides with activity against Mtb, are inactive against *M. smegmatis*^46,47^. The development of a double auxotrophic Mtb strain, Mtb H37Rv mc^2^6206^48^, and more recently an MDR triple auxotrophic Mtb strain, Mtb H37Rv mc^2^7901 and mc^2^7902^49^, which are suitable for use in Biosafety II levels, offer a solution to overcome the safety and accessibility issues associated with limited Biosafety-III space availability required for cultivating Mtb. These auxotrophic Mtb variants, which also more closely resemble the wildtype strain, are already helping to speed up Mtb drug discovery pipelines^50^.

Mtb treatment is highly disease context specific, and antimicrobial resistance (AMR) in TB is compounded by several biological features of Mtb. These include the potential localisation of Mtb cells inside macrophages, the lipid-rich nature of the Mtb cell wall, which contributes to low permeability and limited access to intracellular molecular targets^47^ and the presence of active multi-drug efflux pumps^51^. Metabolically active and replicating forms of TB (active TB), including in drug susceptible-TB, and drug resistant-TB, can be treated by the WHO-recommended standard chemotherapy^1^. However, treatment is more challenging in latent TB infection (LTBI), where an immune response to Mtb antigens occurs in the absence of clinical symptoms^52^. LTBI is commonly treated using a short course including RPT and INZ (3 months), RIF (4 months), INZ and RIF (3 months), INZ (6 or 9 months)^53^. Despite these options, therapy remains difficult because dormant bacteria may persist intracellularly. Anti-TB agents must therefore cross multiple biological barriers, including host cells, to reach intracellular bacteria and permeabilise the mycobacterial cell envelope within infected macrophages^54^. Together, these factors make anti-TB drug discovery particularly challenging.

These shortcomings in current treatment options and the growing AMR challenge underscore the critical need for new, effective TB therapies to overcome the current limitations of therapy, mainly aimed at reducing treatment time and increasing efficacy. Therefore, novel anti-TB candidates are urgently required, particularly those that focus on compounds with a lower likelihood of resistance development, and solutions which can shorten treatment duration, thus facilitating patient compliance.

In this context, this review explores the potential of antimicrobial peptides as alternatives and/or adjuvants in the fight against drug-resistant TB. It focuses on anti-TB peptides described in the literature with a particular attention to those reported in the antimicrobial peptide database (APD6)^55^, with the aim of identifying promising templates, clarifying the strength and limitations of the available experimental evidence, and outlining realistic pathways for their further development for TB treatment.

## Antimicrobial peptides: an alternative/adjuvant for TB treatment

**Fig. 2:**
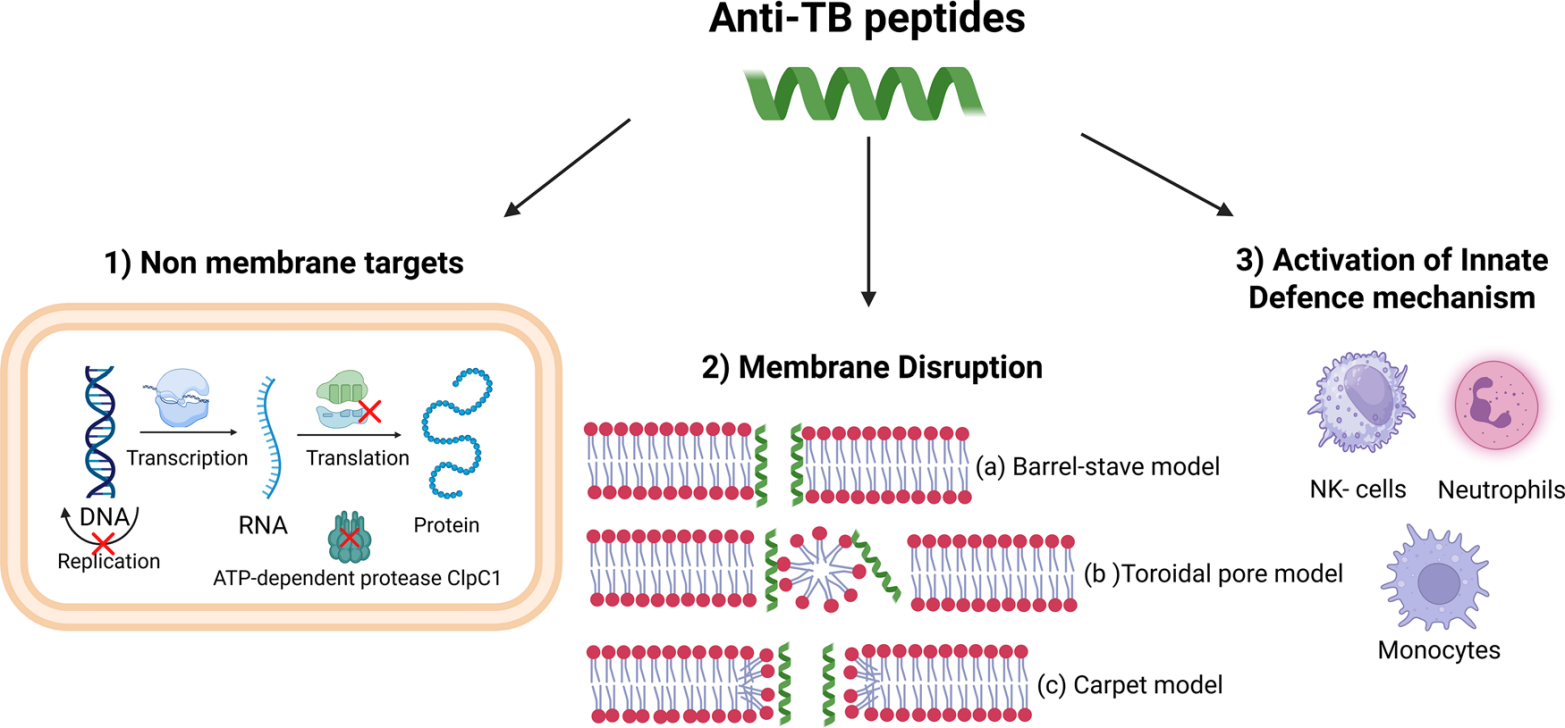
Mode of action of anti-TB peptides. Anti-TB peptides target bacterial cells in three different ways. **1** Non-membrane targets: targeting various intracellular machinery and processes like DNA replication^124^ and translation^121^. **2** Majority of anti-TB peptides act on the cell membrane^72,92,103,110^. They disrupt the membrane in either of three different ways; (a) barrel-stave model: peptides (referred to as staves) are placed in a barrel like ring within the membrane creating pores with their hydrophobic side facing towards the membrane and hydrophilic side making the pore lining (b) toroidal pore model: the attached peptides intercalate with the membrane lipids inclining the lipid monolayer through the pores, so that both the peptides and the head groups face towards the centre of the pore (c) carpet model: peptides lineup in parallel with surface of the membrane, that generates an extensive layer which increase the surface tension that leads to membrane disruption. **3** Peptides can also activate the innate defence mechanism^68^ by disrupting bacterial membranes through electrostatic interactions, leading to cell lysis and targeting intracellular machinery, thereby killing the pathogen. This image was created using BioRender.

Antimicrobial peptides (AMPs) are a group of naturally occurring small peptides synthesised by living organisms as a defense mechanism^56^. These can also include synthetic variants of natural AMPs^57^ or those identified via deep learning^58,59^. These peptides have gained significant attention due to their activity against Gram-positive bacteria^60,61^ and Gram-negative bacteria^60^, different fungal species^62,63^ and viruses^64^. In addition, AMPs function by multi-modes of action (see Fig. 2), including disrupting the cell membrane^65^, interfering with intercellular components^66,67^ and activating the innate immune system^68^, with generally non-specific targets, thereby imposing a low likelihood of resistance development^69,70^. These remarkable properties make AMPs a promising therapeutic regimen against hard-to-treat pathogens, especially MDR variants.

### Anti-TB peptides

The APD6^55^ (available at Antimicrobial Peptide Database) can be considered as a reference standard for AMP research as it offers a curated repository of experimentally validated natural, synthetic and machine learning predicted AMPs, with regularly updated activity data and advanced search tools to accelerate peptide discovery^55^. As of September 2025, there were 5680 peptides described in APD6, reported from six different kingdoms of life. These AMPs are classified based on: (1) source: 423 from bacteria, 5 from archaea, 8 from protists, 29 from fungi, 270 from plants, 2610 from animals, 329 predicted and 1733 synthetic AMPs, (2) activity: anti-bacterial peptides, anti-biofilm peptides, anti-MRSA peptides, anti-TB peptides, anti-endotoxin peptides, anti-toxin peptides, anti-viral peptides, anti-HIV peptides, anti-fungal peptides, anti-candida peptides, anti-parasitic peptides, anti-malarial peptides, anti-cancer peptides, anti-diabetic peptides, wound healing peptides, chemotactic peptides, anti-inflammatory peptides, spermicidal peptides, insecticidal peptides, ion channel inhibitors, protease inhibitors, anti-oxidant peptides, surface immobilised peptides, two-chain peptides, synergistic peptides, (3) structural properties: α-helix, β-sheet, both α-helix and β-sheet and linear, and (4) amino acid-rich species: proline-rich AMPs, tryptophan and arginine rich AMPs, histidine rich AMPs and glycine rich AMPs^55^.

Among the 5680 AMPs described in APD6 database, a mere 26 have been reported to exhibit anti-mycobacterial activity. Out of these, only 22 peptides have been experimentally reported to have anti-TB activity (Table 1). Of the other four peptides reported as anti-TB, Lariatin B^71^ has only been shown to be active against the non-tuberculous mycobacterium, *M. smegmatis*, with no reported activity against Mtb. Human RNase 5 (angiogenin) shows no detectable anti-mycobacterial activity. Similarly, RNase 7^72^ lacks evidence of activity against Mtb and has only been reported to exhibit activity against *Mycobacterium vaccae*. Mouse angiogenin has not been evaluated against mycobacterial species, and its anti-mycobacterial activity remains unelucidated. These observations highlight that several peptides classified as anti-TB agents are supported primarily by activity against non-tuberculous mycobacteria rather than direct evidence against Mtb.

**Table 1:** Anti-TB peptides active against Mtb are reported in the APD6 database

Peptide	Length (AA)	Sequence	Source	MIC Reported	MIC converted to µM	Model type (in vitro/in vivo)	Strain used for the study	Selectivity index	Mechanism	References
Angie1^+^	17	KAINTFIHGNKISIKAI	Computationally identified and modified based on natural derivatives	Not reported	Not reported	in vivo (zebrafish embryo model)	Mtb H37Rv (ATCC 27294)	Not reported	Not elucidated	a, b^75^
B1CTcu5^+^	21	LIAGLAANFLPQILCKIARKC	*Clinotarsus curtipes*	12.5 µg/mL	5.54	in vitro	Mtb H37Rv (ATCC 25618)	Not reported	Morphological changes in the mycobacterial cell wall	a^77^, b^46^
Clovibactin^+^	8	FLKSNALL	*Eleftheria terrae* ssp. carolina	0.5–1 µg/mL	1.1	in vitro	Mtb mc^2^6020	Not reported	Inhibit cell wall synthesis	a, b^80^
Enterocin AS-48^+^	70	MAKEFGIPAAVAGTVINVVEAGGWVTTIVSILTAVGSGGLSLLAAAGRESIKAYLKKEIKKKGKRAVIAW	*Enterococcus faecalis* S-48; human microbiota, gut, symbiont bacteria	32–64 μg/mL	8.92	in vitro	Mtb H37Rv	Not reported	Depolarisation and disrupt the morphology of the bacteria	a^81^, b^82^
Human granulysin^+^	74	GRDYRTCLTIVQKLKKMVDKPTQRSVSNAATRVCRTGRSRWRDVCRNFMRRYQSRVTQGLVAGETAQQICEDLR	*Homo sapiens*	#10.1 µM	10.1	in vitro	Mtb H37Rv	Not reported	Altering the membrane integrity	a^83^, b^84^
hBD10^+^	35	RECRIGNGQCKNQCHENEIRIAYCIRPGTHCCLQQ	*Homo sapiens*	Susceptible 11.8 µM- MDR 5.9 µM	11.8	in vitro	Mtb H37Rv (ATCC 27294), Mtb MDR strain	Not reported	Not elucidated	a, b^91^
hBD consensus^+^	32	AAHLPAEFTPAVHASLDKFLASVSTVLTSKYR	*Homo sapiens*	Susceptible 8.8 ± 3.3 µM, MDR- 25.9 µM	12.1	in vitro	Mtb H37Rv (ATCC 27294), Mtb MDR strain	Not reported	Not elucidated	a, b^91^
HBA (111-142) ^+^	32	KKCWNGGRCRKKCKENEKPIGYCRNGKKCCVN	*Homo sapiens*	Not identified	Not identified	in vitro	Mtb H33rV	Not reported	Membrane permeabilisation	a, b^92^
Kitamycobactin^+^	16	GFGRIKADQLVGRLIP	*Kitasatospora sp*.	0.06 µg/mL	0.03	in vitro	Mtb H37Rv, Mtb mc^2^6020	HepG2 = 1666.7, NIH/3T3 = > 1666.7	Target the mycobacterial ClpP1P2C1 protease.	a, b^95^
Lariatin A^+^	18	GSQLVYREWVGHSNVIKP	*Rhodococcus jostii K01-B0171*	0.39 µg/mL for Mtb, 3.13 µg/mL for *M. smegmatis*	0.01	in vitro	Mtb H37Rv	Not reported	Not elucidated	a^98^, b^71^
Lassomycin^+^	16	GLRRLFADQLVGRRNI	*Lentzea kentuckyensis* sp.	0.8–3 µg/mL	1.59	in vitro	Mtb mc^2^6020	HepG2/NIH 3T3 = 117–438	Target the ATP-dependent protease ClpC1	a, b^96^
Laterosporulin 10^+^	53	ACVNQCPDAIDRFIVKDKGCHGVEKKYYKQVYVACMNGQHLYCRTEWGGPCQL	*Brevibacillus* sp.	0.5 µM	0.5	in vitro	Mtb H37Ra, Mtb H37Rv	Not reported	Membrane-permeabilising peptide	a^102^, b^103^
LL- 37 (Leucine-Leucine 37) ^+^	37	LLGDFFRKSKEKIGKEFKRIVQRIKDFLRNLVPRTES	*Homo sapiens and Pan troglodytes*	*5 µg/mL	1.12	in vitro and in vivo (mouse model)	Mtb H37Rv (ATCC) and an MDR strain	Not reported	Disrupts the cell wall of intra- and extracellular Mtb	a^104^ b^108^
Streptomycobactin+	20	VIITVLLVRAVLATVRIERV	*Streptomyces* sp.	0.03 µg/mL	0.01	in vitro	Mtb H37Rv, Mtb mc^2^6020	HepG2 = ≥533 NIH/3T3 = ≥ 533	Not elucidated	a, b^95^
SyCPA 63^+^	5	KRIRL	*Bacillus thuringiensis* plasmid BMB171	6 µg/mL	8.76	in vitro	Mtb H37Rv	TC₅₀ > 100 µg/mL (highest concentration tested)	Depolarising the bacterial membrane	a, b^110^
Teixobactin^+^	11	FISQIISTARI	*Eleftheria terrae*	0.125 µg/mL	0.1	in vitro	Mtb	Not reported	Inhibits cell wall synthesis by binding to a highly conserved motif of lipid II and lipid III	a, b^111^
VpAmp 1.0^+^	19	LPFFLLSLIPSAISAIKKI	*Vaejovis punctatu*	17.4 ± 6.4 µM	23.8	in vitro	Mtb H37Rv and an MDR strain	Not reported	Not elucidated	a, b^114^
VpAmp 2.0^+^	25	FWGFLGKLAMKAVPSLIGGNKSSSK	*Vaejovis punctatu*	21.4 ± 7.5 *µ*M	28.9	in vitro	Mtb H37Rv and an MDR strain	Not reported	Not elucidated	a, b^114^
Cacaoidin^0^	23	SSAPCTIYASVSASISATASWGC	*Streptomyces cacaoi* CA-170360	32 µg/mL	14.41	in vitro	Mtb H37Ra	Not reported	Dual mode of action (binds to lipid II_PGN_ and inhibits cell wall transglycosylases)	a^116^,b^118^
Micrococcin P1^0^	14	SCTTCVCTCSCCTT	*Staphylococcus epidermidis* strain, 115; *Staphylococcus equorum* WS 2733	32–63 nM	0.063	in vitro	Mtb H37Rv	>500	The peptide targets the bacterial ribosome and inhibits translocation	a^119,120^, b^121^
Griselimycin^0^	10	VPSLPLVPLG	*Streptomyces*	1–6.2 µg/mL under various conditions	6.25	in vitro and in vivo (mice model)	Mtb H37Rv	Not reported	Target DnaN and thereby inhibit DNA replication	a^122^, b^124^
Pantocin wh-1^0^	16	VMWCYVFGYGFNCAVW	*Pantoea dispersa* W18	7.5 μg/mL	3.85	in vitro and in vivo (mice model)	Mtb H37Ra	Not reported	Still under investigation	a, b^125^

^a^Reference for peptide identification, ^b^reference for anti-TB activity. ^#^minimum inhibitory concentration (MIC) determined using a synthetic fragment derived from the parent peptide; *MIC determined using a synthetic peptide; ^+^cationic antimicrobial peptides (AMPs); 0: neutral peptides. Selectivity index (SI) values were extracted directly from the original studies when reported; otherwise, SI was calculated as TC_50_ (50% toxic concentration), IC_50_ (50% inhibitory concentration), or EC_50_ (50% effective concentration) divided by MIC. Cytotoxicity or haemolysis values used for SI calculation were obtained from mammalian models, including red blood cells (RBCs), NIH/3T3 fibroblasts, and HepG2 cells, as described in the original studies. MIC values were converted to µM using the formula: MIC (µM) = [MIC (µg/mL) × 1000]/molecular weight (g/mol).

Of these 22 anti-TB peptides, 18 are cationic, and four have a net zero charge. Additionally, 15 peptides are ≤40 amino acids long^55^. AMPs with shorter sequences are often considered promising therapeutic candidates because shorter peptide length can improve target selectivity and specificity, while also lowering manufacturing complexity and production costs compared with larger biologics and longer therapeutic peptides^73,74^. Although there is very limited information in the literature regarding the mode of action of some of these peptides, an overview of the confirmed and/or suggested modes of action for these known anti-TB AMPs is highlighted in Fig. 2.

### Mechanisms, structural and functional features of anti-TB peptides

#### Cationic anti-TB peptides

**Angie1:** Angie1 is a 17-amino acid (AA) synthetic peptide, identified using computational tools and chemically modified based on human Angiogenin^75^. It is classified under the ribonuclease A (RNase A) superfamily, similar to its parent peptide Angiogenin^76^. Angie1 exhibited dose-dependent activity against extracellular Mtb, with near-maximal inhibition observed at 100 µM. It also showed activity against intracellular Mtb and was non-toxic to human macrophages and zebrafish embryos (in vivo). However, a formal minimum inhibitory concentration (MIC) was not determined, and the mode of action and intracellular targets remain unidentified^75^.

**B1CTcu5:** B1CTcu5 is a synthesised 21 AA peptide originally obtained from the skin secretions of the Indian frog *Clinotarsus curtipes*^77^. It is associated with the Brevinin-1 family of AMPs, harbouring a unique C-terminal cyclic region called “rana box”, which is an intradisulphide bond produced by the oxidation of positively charged C-terminal and 7th cysteine residue. With an MIC of 12.5 µg/mL against Mtb, B1CTcu5 was capable of successfully eradicating intracellular Mtb without affecting macrophages in vitro. The mechanism of action of B1CTcu5 against Mtb involves modifications in the bacterial cell wall, including cavitation and thinning^46^. Another peculiarity of the peptide is its hydrophobic nature (65%), which plays a major role in transporting the peptide from aqueous to hydrophobic conditions and increasing its affinity towards the membrane lipid acyl chains, making a lipid-peptide complex^46^. This might generate a rearrangement in bacterial cell membranes, resulting in thinning, pore formation, altered curvature and perturbation. The translocation of the peptide through the membrane into the cytoplasm may allow its interaction with potential intracellular targets^78^, though these have not yet been identified. Moreover, another study^79^ revealed that B1CTcu5 needs its entire structure/length for its bactericidal action.

**Clovibactin:** Clovibactin is a depsipeptide (peptide with both amide and ester bonds) with 8 AAs isolated from uncultured soil bacteria, *Eleftheria terrae* ssp. carolina^80^. It contains two D-amino acids in its linear N-terminal region and an unusual D-3-hydroxy-asparagine residue in its depsi-cycle^80^. It showed an MIC of 0.5–1 µg/mL against Mtb mc^2^6020. It also exhibited low cytotoxicity against mammalian cell lines (NIH/3T3, Hep-G2)^80^. Clovibactin functions by inhibiting bacterial cell wall synthesis by tightly binding pyrophosphate groups of essential cell wall precursors like lipid II, lipid III, and C55-PP^80^. This binding is specific to bacterial membranes containing lipid-anchored pyrophosphate, which results in the formation of supramolecular fibrils on the membrane surface. These fibrils inhibit access to precursors, thereby blocking cell wall synthesis. This novel approach avoids resistance by creating a physical barrier that entraps precursors in fibrillar assemblies^80^. Clovibactin also exhibits structural similarities with Teixobactin, another anti-TB peptide reported in the APD6 database^55^.

**Enterocin AS-48:** Enterocin AS-48 (AS48) is a ribosomally synthesised and post-translationally modified peptide (RiPPs) produced by *Enterococcus faecalis*^81^. It is a 70 AA peptide with an alpha helical structure that interacts with the cell membrane. It also exhibits a broad spectrum of activity, including against Mtb^82^ and other Gram-positive and Gram-negative bacteria^82^. AS48 has an MIC of 32–64 μg/mL against Mtb H37Rv and several Mtb clinical isolates. It also showed synergism with the conventional anti-TB drug EMB with a fractional inhibitory concentration index (FICI) of 0.09375. AS48 gave rise to depolarisation of the mycobacterial membrane, followed by the alteration of the bacterial morphology. It was found to have low cytotoxicity against human and mouse cell lines (THP-1, MH-S and J774.2) at the doses required for inhibition of the bacterial growth^82^. Additionally, AS48-EMB showed stronger synergism against intracellular Mtb during macrophage (Raw 264.7) infection assays and with the FICI values for intracellular activity being identical to that observed in the in vitro assays.

**Human granulysin:** Granulysin is a 74 AA human polypeptide^83^ with anti-TB activity^84^. It is present in the cytoplasmic granules of T lymphocytes and Natural killer cells (NK cells) of *Homo sapiens*^83^. It belongs to the saposin-like protein (SAPLIP) family, with Cys-rich groups and is composed of three disulphide bonds, which are essential for antimicrobial activity. Granulysin alone was active against extracellular Mtb, and when combined with perforin, a pore-forming protein that creates pores on cell membranes, its viability against intracellular bacteria was improved^84^. Furthermore, granulysin has been reported to be toxic against Mtb by destroying the cell wall and interrupting the lipid metabolism of Mtb to generate osmotic shock^84^. The synthetic peptides GranF1 and GranF2 synthesised from granulysin, retained the antimicrobial activity, notably, against both drug-susceptible-TB and MDR-TB^85^. In addition, synthetic peptide Gr-1C derived from residues 25–50 of granulysin displayed a low MIC of 10.1 μM in comparison with other synthetic variants (Gr-1L, Gr-2 and Gr-3)^86^.

**hBD10 and hBD*consensus:*** Human β-defensins (hBDs) are the most explored human-derived cationic AMPs^87,88^. Although computational and bioinformatic analysis of the human genome has identified numerous hBDs^89,90^, the biological activities of fewer than half have been experimentally verified^91^. Both the hBD10 (35 AA) and hBD*consensus* (32 AA) are synthetic peptides folded in vitro. hBD*consensus* was designed by aligning sequences related to the defensin_beta_2 domains (Pfam, PF00711), incorporating both experimentally characterised and predicted hBDs sequences, while hBD10 was designed based on a 35-residue peptide from the *DEFB110* human gene^91^. The structured region of hBD*consenus* is composed of triple-stranded antiparallel β sheets, stabilised by three disulfide bridges like all hBDs. These compounds play a vital role in innate defense mechanisms not only by directly killing the pathogen but also by hiring defense cells through chemotaxis, stimulating angiogenesis, wound healing and connecting the innate and adaptive immune system. Both peptides impair the growth of wild-type Mtb and MDR-Mtb. hBD10 showed moderately greater inhibition activity towards MDR-Mtb (MIC 5.9 µM) compared to the wild type strain (MIC 11.8 µM), and hBD*consensus* was highly active against the wild type strain (MIC 8.8 ± 3.3 µM)^91^. Both peptides exhibited better activity against susceptible Mtb in comparison with RIF and EMB, while they revealed stronger inhibitory activity than INH, RIF and EMB against the MDR-Mtb^91^. Although neither peptide was cytotoxic to human erythrocytes, their mechanism of action and intercellular targets remain unknown.

**HBA (111–142):** HBA (111–142) is a naturally occurring C-terminal 32-mer fragment of alpha-haemoglobin identified from a spleen-derived antimicrobial peptide library^92^. It is an amyloidogenic, 30-residue peptide enriched in cationic amino acids (Lys, Arg, and His), which become positively charged under acidic conditions and promote the formation of β-sheet-rich, cationic amyloid-like fibrils^92^. This likely induces conformational changes that facilitate insertion into the bacterial membrane, potentially explaining the peptide’s increased antibacterial activity under acidic conditions^93^. Additionally, HBA (111–142) contains an alternative arrangement of hydrophilic and hydrophobic residues, an ideal structure suitable for binding the bacterial membranes^94^. It is active in both non-fibrillar (solubilised form) and fibril form. It disrupts the bacterial cell membrane by integrating into the lipid bilayer, leading to membrane leakage and cell death, whereas under acidic conditions, it forms amyloid-like fibrils that can act as a bacterial aggregator. Its cationic, β-sheet-rich fibrils adhere to bacterial surfaces and stimulate clumping, thereby facilitating immune recognition and phagocytic clearance^92^. Although both solubilised and fibril forms killed extracellular Mtb and displayed low cytotoxicity^92^, their MIC and action against intracellular Mtb remain undefined.

**Kitamycobactin:** Kitamycobactin is a novel 16 AA anti-TB peptide obtained from screening uncultured bacteria against Mtb and is produced by *Kitasatospora* sp^95^. It is an analogue of lassomycin reported earlier in 2014^96^. It displayed a potent MIC of 0.06 µg/mL against Mtb. It also exhibited an MIC range of <0.1–1 µg/mL against different species within the *Mycobacterium* genus. Kitamycobactin has low cytotoxicity against mammalian cell lines (NIH/3T3 and HepG2; TC_50_ = 100 µg/mL) and has a great therapeutic window against Mtb^95^. It targets ClpP1P2C1^95^, an important protease of mycobacteria, which plays a crucial role in protein degradation and growth of Mtb^97^. However, its activity against intracellular Mtb needs further investigation.

**Lariatin A:** Lariatin A is an 18AA anti-TB peptide isolated from *Rhodococcusjostii* K01-B0171^98^. Lariatin A has a ‘lasso’ structure where the C-terminal tail (Trp-9-Pro-18) loops back and passes through the N-terminal 8-residue macrolactam ring connected by an iso-peptide bond established through the amino group of Gly-1 and the carboxylic acid side chain of Glu-8^98^. Lariatin A is composed of three main hydrophobic regions, and it is classified into the class II lasso peptide group as it lacks disulfide bonds^99^. The topology of this peptide group is solely maintained by the steric interaction between amino acids^100^. Lariatin A exhibited an MIC of 0.39 µg/mL for Mtb^71^. Molecular simulations revealed that Lys-17 in the C-terminal tail of Lariatin A could play a vital role in antimicrobial activity^101^. Although it is proposed to target the mycobacterial cell and inhibit the cell wall synthesis^98^ its mode of action still remains elusive.

**Lassomycin:** Lassomycin is a potent antibacterial compound obtained from the extracts of the bacterium, *Lentzea kentuckyensis* sp^96^. The peptide consists of 16 AA structured as a lasso, in which an eight-residue ring resembles the loop, and the other residues form the spoke. The peptide eradicated bacteria selectively by targeting ClpC1 without affecting the associated hexameric AAA ATPase, showing an MIC of 0.8-3 µg/mL against Mtb. Even at low concentrations, the peptide can permeate Mtb and bind to an acidic N-terminal pocket on ClpC1, a crucial enzyme for viability in mycobacteria, a rare target, which needs further investigation. The bacterial cell death could be caused by either the prevention of ClpC1-mediated lysis of essential protein or by an excessive increase in ClpC1’s activity, for instance, high-rate ATP hydrolysis or excessive protein unfolding^96^. This peptide did not lyse erythrocytes and had low cytotoxicity (IC_50_, 350 μg/ml) against human NIH/3T3 and HepG2 cells, promising a safe therapeutic window^96^. Furthermore, the threaded form of lassomycin is essential for its antimicrobial activities, as the unthreaded version of the lasso peptide and lassomycin-amide do not show any biological activities^96^.

**Laterosporulin10:** Laterosporulin10 is a heat-stable 53 AA cysteine-rich peptide produced by *Brevibacillus* sp^102^. This class IId bacteriocin exhibits a structural resemblance, although having a low sequence similarity, to human defensins^102^. The peptide is hydrophobic, important for its interaction with the bacterial cell membrane. Like other bacteriocins, and other peptides from the laterosporulin family, it functions by disrupting and permeabilising the bacterial cell wall, followed by cellular metabolism^103^. The peptide has shown activity against intracellular Mtb residing inside the phagosomes (RAW 264.7). The bacteriocin was more active against the Mtb H37Rv strain than RIF and PZA. Additionally, it displayed a synergistic activity with RIF (FICI = 0.275)^103^. The deviation in the ATP levels and the NAD/NADH and NADP/NADPH ratios that lead to bacterial death show that the cell wall of Mtb was also disintegrated by the presence of this peptide. Furthermore, the AMP was non-cytotoxic and non-haemolytic even at high concentrations, making it a potential candidate for TB treatment.

**LL-37 (Leucine-Leucine 37)**: LL-37 is the only human cathelicidin identified as an AMP. The precursor hCAP-18 (human cationic antimicrobial protein, ~18 kDa) is cleaved by proteases to release the active peptide LL-37^104^. The three-dimensional structure of LL-37 reveals the presence of a distinct long helix covering residues 2–31 followed by a disorganised C-terminal. The peptide has an amphipathic structure, where the hydrophobic surface is disrupted by a hydrophilic Ser-9 and a helical bend between residues Gly-14-Glu-16. LL-37 also has a unique aromatic ring formed by Phe-5, Phe-6, Phe-17 and Phe-27 that might be essential for the association of the amphipathic helix with the anionic charged components of the bacterial cell wall^105^. The peptide functions by interacting with various components of the bacterial membrane (lipopolysaccharide, peptidoglycan, phospholipids and lipoteichoic acid), thereby inhibiting bacterial growth by interfering with the synthesis of the above-mentioned components or by disrupting membrane integrity^106,107^. LL-37 functions in host cells by the activation of Toll-like receptors on human macrophages via a Vitamin D-dependent pathway^107^, however, the MIC of synthetic LL-37 against Mtb (5 µg/mL) was reported by Santiago et al.^108^. This study further demonstrated that LL-37 produced a transient reduction in lung Mtb burden, followed by regrowth and no significant improvement in lung pathology. Although LL-37 has a significant role in immunomodulation and eradication of numerous pathogens, including Mtb, it is highly toxic to human cells^109^ and its chemical synthesis is not cost-effective in comparison with other peptides reported in ADP6 database^55^.

**SyCPA63:** SyCPA63, also known as ‘Thurinsyn’ is a synthetic peptide identified using the synthetic-bioinformatic natural product (syn-BNP) approach and chemically synthesised based on the predictions obtained from bioinformatics algorithms^110^. SyCPA63 (syn-BNP cyclic peptide antibiotics- SyCPA63) is the shortest anti-TB peptide of the APD6 database, with 5 AA with macrocycles. SyCPA63 contains cyclisation through nucleophilic side-chain^110^. It inhibited the growth of Mtb at 6 μg/mL and functions by depolarising the bacterial membrane^110^. Although it was not toxic to HeLa cells^110^, its cytotoxicity assessment against BEAS-2B and intracellular macrophage assays are essential to evaluate its efficacy.

**Streptomycobactin:** Streptomycobactin is a 20 AA anti-TB peptide produced by *Streptomyces* sp^95^. It showed great activity against Mtb with an MIC of 0.03 µg/mL. It exhibited a toxic concentration 50% (TC_50_) of 16 to 32 μg/mL against HepG2 and NIH/3T3 cell line^95^. However, the mechanism of action of streptomycobactin needs further investigation.

**Teixobactin:** Teixobactin is an 11 AA peptide isolated during screening of the uncultured bacteria *Eleftheria terrae* using the iChip culturing technique^111^. It is a cyclic undecapeptide consisting of four D-aminoacids *N-Me*- _D_-Phe^1^, _D_-Gln^4^-*allo*-Ile^5^, and _D_-Thr^8^, and a unique _L_-*allo*-enduracididine amino acid^112^. This AMP is a cell wall inhibitor that acts by binding to the lipid II precursor for peptidoglycan biosynthesis and lipid III precursor for teichoic acid biosynthesis. Although teixobactin gained a lot of attention for its high activity against multidrug-resistant *Staphylococcus aureus* (MRSA) and Mtb, there are no ideal synthetic routes for teixobactin and its analogues that retain the unusual amino acid L-*allo*-enduracididine. Certain research groups revealed the synthesis of a teixobactin analogue in which _L_-*allo*-enduracididine was replaced by _L_-arginine and _L_-lysine. These novel analogues showed an identical antimicrobial profile to teixobactin against *Staphylococcus aureus*^113^, however, there is no information about the activity of teixobactin analogues against both drug-susceptible and drug-resistant TB.

**VpAmp1.0 & VpAmp2.0:** VpAmp1.0 (19 AA) and VpAmp2.0 (25 AA) are two cationic non-disulphide bound (NDBPs) peptides obtained from the venom glands of *Vaejovis punctatus*, a Mexican scorpion^114^. These peptides are active against Mtb and MDR-TB. VpAmp1.0 showed an MIC of 17.4 ± 6.4 μM (Mtb) and 4.8 ± 1.5 μM (MDR-TB) μM. VpAmp2.0 displayed an MIC of 21.4 ± 7.5 μM (Mtb) and 30.5 ± 9.5 μM (MDR-TB) respectively^114^. One of the major challenges with scorpion-derived AMPs is their cytotoxic effect on human cells, as they interact with cell membranes non-specifically rather than binding to membrane receptors^115^. To study the structural and biological importance of these peptides, two variants VpAmp1.1 and VpAmp2.1 were synthesised and evaluated along with the parent compounds. VpAmp1.1 lacks the dipeptide Leu-Pro from the N-terminal region of VpAmp1.0, and VpAmp2.1 lacks the Ser triad at the C-terminal of VpAmp 2.0. Peptide VpAmp1.0 was the most haemolytic, while the deletion of N-terminal Leu-Pro dipeptide from VpAmp1.0 made VpAmp1.1 four times less haemolytic. Contrarily, modification of VpAmp2.1 by the removal of hydrophilic Ser triad from VpAmp2.0 made it more haemolytic than the parent peptide^114^. Both the variants were active against Mtb and MDR-TB, like their parent peptide. VpAmp1.1 displayed an MIC of 5.4 ± 1.7 μM (Mtb) and 8.6 ± 3.3 μM (MDR), whereas VpAmp2.1 exhibited an MIC of 13.6 ± 5.3 μM (Mtb) 8.5 ± 2.6 μM (MDR). However, the mode of action and intercellular targets of these peptides are still unknown.

#### Neutral anti-TB peptides

**Cacaoidin:** Cacaoidin is a 23 AA glycosylated lantibiotic isolated from a *Streptomyces cacaoi* strain. It is the first member of the new family of RIPPs entitled lanthidins^116^. Detailed analysis of the biosynthetic gene cluster categorised it as the first class V lanthipeptide^117^. It is characterised by the presence of N,N-dimethylation of the N-terminal alanine, glycosylated tyrosine residue, an extraordinary N,N-dimethyl lanthionine and several D-amino acids^116^. It is the first natural compound demonstrated to have a dual mode of action, combining direct binding to lipid II_PGN_ with the inhibition of cell wall transglycosylases^118^. Although an MIC of 32 µg/mL is reported against Mtb^118^, further investigation is required to verify its effectiveness towards intracellular Mtb.

**Micrococcin-P1(MP1):** Micrococcin-P1(MP1) is an anti-TB peptide isolated from *Staphylococcus* epidermidis^119^ and Staphylococcus *equorum* WS 2733^120^, with proven activity against Mtb^121^. MP1 is a 14 AA peptide highly active against Mtb with an MIC of 32–63 nM, low cytotoxicity and a selectivity index >500^121^. It also showed activity against intracellular Mtb growing inside RAW264.7 macrophages. It functions by targeting both the N-terminus proline loop of L11 and 23S rRNA^121^. It inhibits both EF-Tu and EF-G mediated steps in the protein translation and thereby hinders protein synthesis in mycobacteria^121^.

**Griselimycin:** Griselimycin is a 10 AA cyclic anti-TB peptide obtained from *Streptomyces* strains^122^. The peptide belongs to the linaridin family of ribosomally synthesised peptides and has a chemical structure similar to cypemycin^123^. It functions by inhibiting DNA polymerase sliding clamp DnaN, resulting in antibacterial activity against Mtb^124^. Synthetic derivatives of griselimycins were developed with increased potency, metabolic stability, and higher exposure towards the target site. The methyl-derivated griselimycin (MGM) and the cyclohexyl-griselimycin (CGM) demonstrated improved properties, with CGM becoming the lead candidate to treat susceptible and resistant Mtb due to its improved activity^124^. CGM also demonstrated potent in vivo bactericidal activity against both actively replicating and non-replicating Mtb. While rare, it is noted that resistant strains can emerge against GM and CGM, through multiplication of *dnaN*, where the higher amplification of *dnaN*, the greater resistance towards griselimycin^124^.

**Pantocin wh-1:** Pantocin wh-1 is a 16 AA peptide and the first cyclic polypeptide obtained from *Pantoea dispersa*^125^. It exhibited in vitro activity against Mtb with an MIC of 7.5 µg/mL and demonstrated in vivo efficacy, although its therapeutic effect did not exceed that of the control drug streptomycin. Its anti-mycobacterial activity was completely eliminated by protease K and partially reduced by trypsin and pepsin digestion, indicating a peptide-dependent mechanism^125^. Pantocin WH-1 also showed high stability across a broad pH range (2–12) and resistance to heat treatment, consistent with cyclic peptides. These findings highlight its therapeutic potential, further optimisation is needed to improve its in vivo efficacy, while its detailed structural characteristics and intracellular targets remain to be fully elucidated^125^.

#### Other anti-TB peptides

Seeing as the APD6 database is manually curated, it does not contain a complete catalogue of all anti-TB AMPs in real time. Additional peptides with documented anti-TB activity that are not yet included in the database are summarised in Table 2 below. Even this expanded list is not exhaustive, but it highlights 20 further peptides that have been identified in the literature

**Table 2:** Other anti-TB peptides reported in the literature show activity against Mtb

Peptide	Length (AA)	Sequence	Source	MIC reported	MIC converted to µM	Model type (in vitro/in vivo)	Strain used for study	Selectivity index (SI)/therapeutic index (TI)	Mechanism	Reference
AK-15	15	AKKKLSRWWLRWWVK	Mycobacteriophage Che12	37.5 μg/mL	18.1	in vitro/in vivo (mice)	Mtb H37Rv, Mtb H37Ra	Not reported	Membrane disruption	a, b^131^
AK 15-6	15	AVKKLLRWWS RWWKK	Mycobacteriophage Che12	18.75 μg/mL	18.1	in vitro/in vivo (mice)	Mtb H37Rv, Mtb H37Ra	Not reported	Membrane disruption and binding to trehalose 6,6′-dimycolate	a, b^131^
αTatM3	11	FGRKKRRPQRR	Synthetic	Mtb = 36.3 µM MDR-Mtb = 15.0 µM	15	in vitro	Mtb H37Ra (ATCC 25177), MDR-Mtb	Mtb =0.23 MDR-Mtb= 0.43	Moderate membrane disruption	a, b^135^
CaDef2.1_G27-K44_	18	GLTRLRRILFRLLLWRTK	*Capsicum annuum*	33.2 µM	33.2	in vitro	Mtb H37Rv (ATCC 27294)	Not reported	Not elucidated	a, b^183^
E50-52	39	TTKNYGNGVCNSVNWCQCGNVWASCNLATGCAAWLCKLA	*Enterococcus faecalis*	0.1 mg/mL	24.24	in vitro/in vivo (mice)	Mtb H37Rv	Not reported	Pore formation	a, b^184^
D-hLF 1–11 (D-enantiomer human lactoferricin 1–11)	11	GRRRRSVQWCA	*Homo sapiens*	100–200 µg/mL	72.75	in vitro	Mtb H37Rv, Mtb H37Ra	**TI = > 40	Membrane permeabilisation	a^174^,b^185^
D-LAK-120-A	25	KKLALALAKKWLALAKKLALALAKK	Synthetic	>256 µg/mL	>94.64	in vitro/ex vivo (THP-1)	Mtb MDR Strain	Not reported	Membrane permeabilisation and access to intercellular bacteria	a^166^,b^150^
D-LAK120-HP13	25	KKALAHALKKWLPALKKLAHALAKK	Synthetic	>256 µg/mL	>92.16	in vitro/ex vivo (THP-1)	Mtb MDR Strain	Not reported	Membrane permeabilisation	a^166^,b^150^
ƔTat1M	11	FGRKKRRQRRR	Synthetic	20.1 mM	20.1	in vitro	Mtb H37Ra (ATCC 25177), MDR-Mtb	Mtb = 0.15 MDR-Mtb = 0.15	Membrane permeabilisation	a,b^135^
ƔTatM4wg	9	FGKKKRQKR	Synthetic	12.8 mM	12.8	in vitro	Mtb H37Ra (ATCC 25177), MDR-Mtb	Mtb = 0.14 MDR-Mtb = 0.23	Membrane disruption	a,b^135^
HCL 2	35	ESTYQGHHTPPVQKGLRYGIILFITSEVFFFAGFF	*Homo sapiens*	15 µg/mL	3.71	in vitro/in vivo (mice)	Mtb H37Rv	Not reported	Disrupt ESAT-6: CFP-10 complex and inhibit both intracellular and extracellular Mtb	a, b^175^
Hepcidin	25	DTHFPICIFCCGCCHRSKCGMCCKT	*Homo sapiens*	Not identified	Not identified	in vitro	Mtb H37Rv	Not reported	Inhibit the release of recycled iron by macrophages and structural damages	a^186^, b^187^
Iztli peptide (IP-1)	19	KFLNRFWHWLQLKPGQPMY	Synthetic	Not identified	Not identified	in vitro/in vivo (mice)	Mtb H37Rv (ATCC 27294), MDR CIBIN99	Not reported	Bactericidal activity with reduced metabolic (ATP-associated) viability; host-directed effects include autophagy activation and TNF-α induction	a^188^, b^189^
Lynronne 1	19	LPRRNRWSKIWKKVVTVFS	Bovine rumen microbiome	292 ± 20 µg/mL	121.62	in vitro	Mtb H37Rv mc^2^6230	**TI = 1.6–6.0	Membrane permeabilisation	a^180^, b^181^
Lynronne 2	20	HLRRINKLLTRIGLYRHAFG	Bovine rumen microbiome	204 ± 6.2 µg/mL	83.7	in vitro	Mtb H37Rv mc^2^6230	**TI = 5.7– > 6.7	Membrane permeabilisation	a^180^,b^181^
Lynronne 2 D_all_	20	HLRRINKLLTRIGLYRHAFG	Bovine rumen microbiome	114 ± 11 µg/mL	46.81	in vitro	Mtb H37Rv mc^2^6230	**TI = 10.7- > 38.5	Non permeabilising peptide	a^180^,b^181^
P15s D_all_	20	KFVRLKIYCRDKNKGRGISF	Bovine rumen microbiome	176 ± 13 µg/mL	72.45	in vitro	Mtb H37Rv mc^2^6230	**TI = 10.1- > 10.9	Membrane permeabilisation	a^180^, b^181^
PK-34	35	PRVIETKVHGREVTGLARNVSEENVDRLAKRWIK	Mycobacteriophage D29	50 µg/ml	12.63	in vitro/in vivo (mice)	Mtb H37Rv (ATCC 27294)	Not reported	PK34, which is trehalose-6,6-dimycolate (TDM), is the enzymatic target	a, b^190^
Protegrins-1	18	RGGRLCYCRRRFCVCVGR	*Sus scrofa*	Not identified	Not identified	in vitro	Mtb H37Rv (ATCC 27294), MDR RM22	Not reported	Presumed membrane disruption	a^191^, b^192^
NZX (plectasin derivative)	40	GFGCNGPWSEDDIQCHNHCKSIKGYKGGYCARGGFVCKCY	*Pseudoplectania nigrella*	6.3 μM	6.3	in vitro/in vivo (mice)	Mtb H37Rv (ATCC 27294)	Not reported	Interact with bacterial membrane but intercellular targets remain undiscovered	a, b^193^

All peptides listed are cationic. ^a^reference for peptide identification, ^b^reference for anti-TB activity. SI (selectivity index) was defined as TC_50_, IC_50,_ or EC_50_ divided by MIC and was calculated only when both cytotoxicity and MIC values were reported within the same study; SI was not calculated when these values originated from different publications or when MIC was not reported. Cytotoxicity data were derived from different mammalian models (RBCs, NIH/3T3, HepG2) **: therapeutic index (TI). MIC (µM) = [MIC (µg/mL) × 1000]/molecular weight (g/mol).

### Descriptive sequence and physicochemical trends among anti-TB AMPs

Several AMPs have demonstrated activity against Mtb, and it is therefore important to determine whether these peptides share common structural or physicochemical features that underpin their efficacy. Identifying and interpreting such shared characteristics can provide a strategic foundation for the rational design or repurposing of AMPs for anti-TB applications.

#### Amino-acid composition and MIC-associated trends

**Fig. 3:**
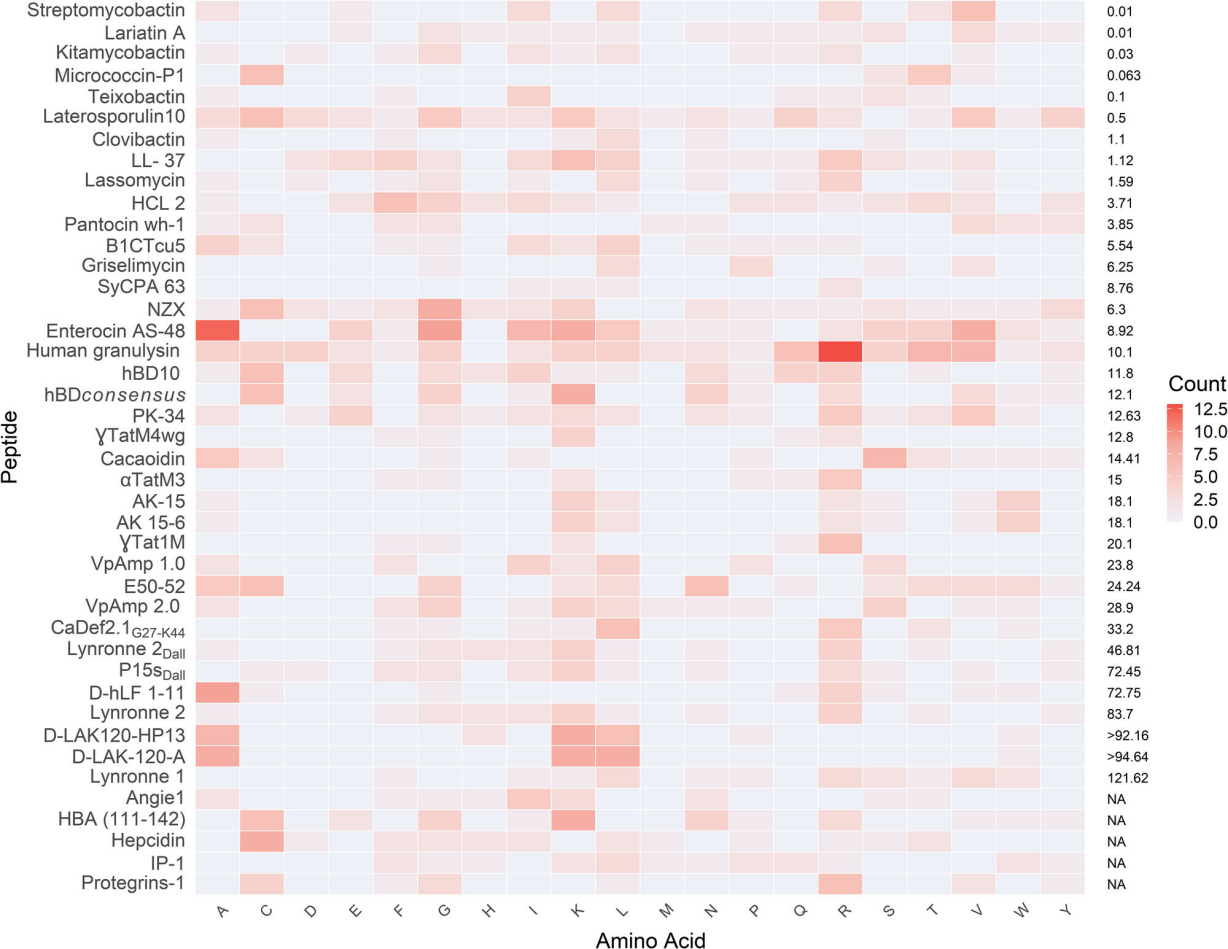
Amino acid (AA) composition heatmap of anti-TB peptides and their reported MICs. The heatmap was generated using data from Tables 1 and 2 and includes 42 anti-TB peptides curated from the APD6 database and published literature. Each row represents a peptide, and each column represents an AA. Colour intensity (light pink to dark red) indicates the frequency of each amino acid within a given peptide. The right-hand column reports MIC values in µM, calculated using the formula described in Tables 1 and 2 for activity against Mtb, with NA indicating peptides for which MIC data are not available. Lower MIC values indicate higher anti-TB activity. This analysis is descriptive and highlights compositional trends; for instance, Ala (A), Leu (L), Arg (R), and Lys (K) are more frequently observed in peptides with lower reported MICs. However, AA composition alone cannot predict peptide activity; it reveals patterns but not the underlying mechanism. This image was created using the ggplot2 package in R-version 4.5.2^194,195^.

**Fig. 4:**
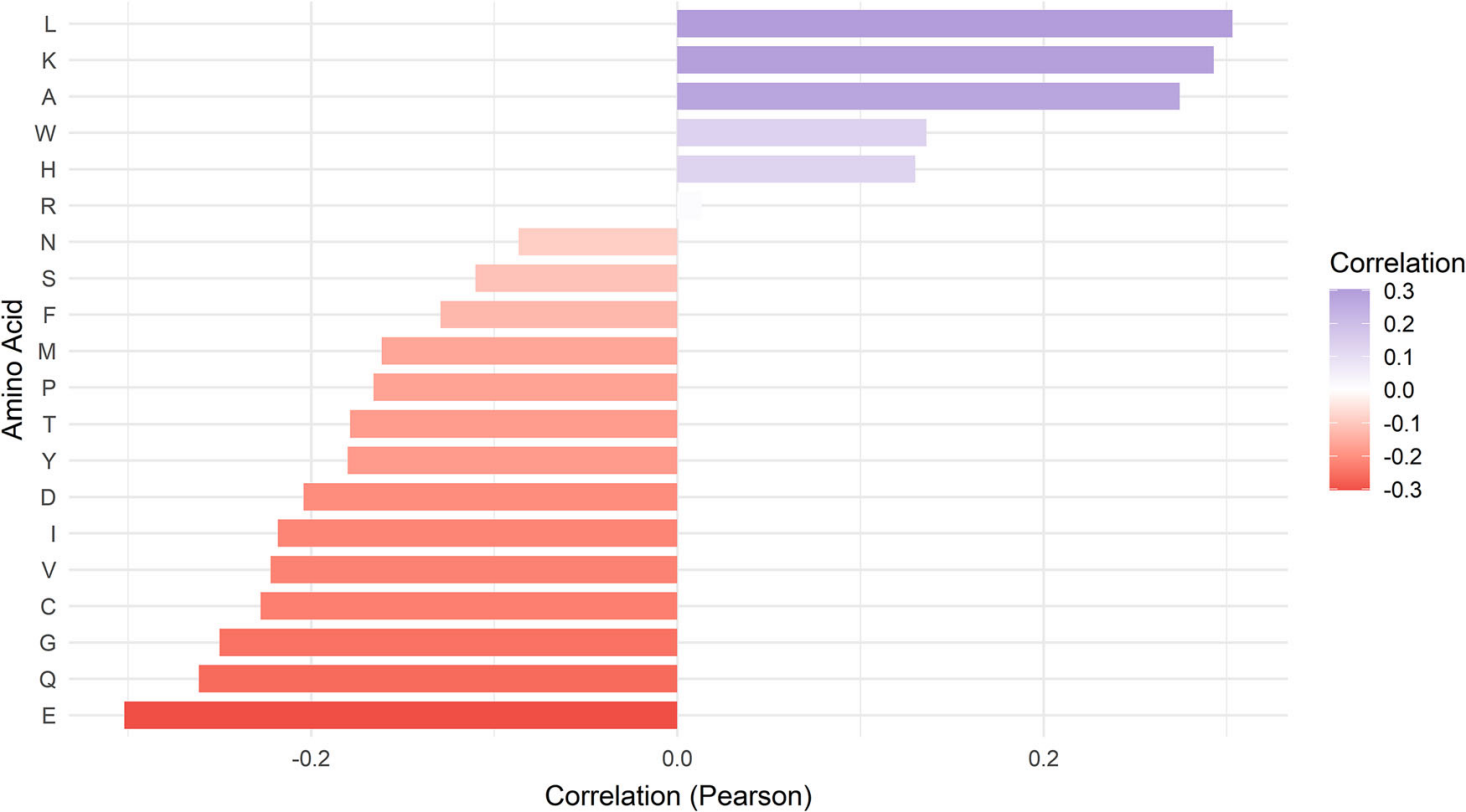
Correlation of amino acid’s frequency and their in vitro antimicrobial activity (MIC). This graph is plotted using data obtained from Tables 1 and 2. Purple indicates a positive correlation with MIC, while red indicates a negative correlation. This analysis is descriptive and does not account for primary sequence order or structural context. For example, Cys(C) residues contribute to disulphide bond formation in certain peptides (e.g., B1CTCu5, human granulysin, and laterosporulin 10); therefore, their functional role cannot be inferred from amino-acid composition alone. Accordingly, these correlations provide an overview of compositional trends observed among reported anti-TB peptides rather than direct or generalisable rules for AMP optimisation. This image was created using the ggplot2 package in R-version 4.5.2^194,195^.

Taking together all the 42 documented anti-TB peptides, i.e., 22 in Table 1 and 20 in Table 2, we observed several recurring trends. Unsurprisingly, most of these peptides are cationic (90.47%), rich in positively charged residues such as Ala (A), Leu (L) Lys (K) and Arg (R) (Fig. 3), which are thought to facilitate interaction with the negatively charged mycobacterial membrane. Ala (A), Leu (L), and Lys (K) are associated with lower MIC values in this dataset, consistent with higher in vitro anti-TB activity (Fig. 4). Despite being essential for peptide structure and function through disulphide bond formation^126^, Cys (C) residues appear to be negatively correlated with activity in Fig. 3. Indeed, cysteine-rich sequences are also prevalent (30.95% i.e., 13/42) within the anti-TB AMP dataset considered in this review, often forming disulphide bonds that might confer structural stability and resistance to proteolytic degradation^127,128^. Notably, several peptides (lassomycin^96^, kitomycobactin^95^ and lariatin A^71^) adopt lasso architectures or β-defensin-like folds, like human defensin-derived peptides. These examples suggest that structural constraints may enhance activity, but only within specific peptide templates. In terms of size, effective anti-TB peptides range from short, cyclic motifs ( ~ 10 residues) to larger, structured peptides ( > 70 residues), though short-to-medium-length peptides (20–40 amino acids) (Tables 1 and 2) are often favoured for synthetic feasibility and reduced immunogenicity^129^. However, these associations are descriptive and template-dependent. It is also important to note that AA/MIC correlations are compositional and do not capture higher-order structural constraints.

#### Descriptive trends in net charge and hydrophobicity

**Fig. 5:**
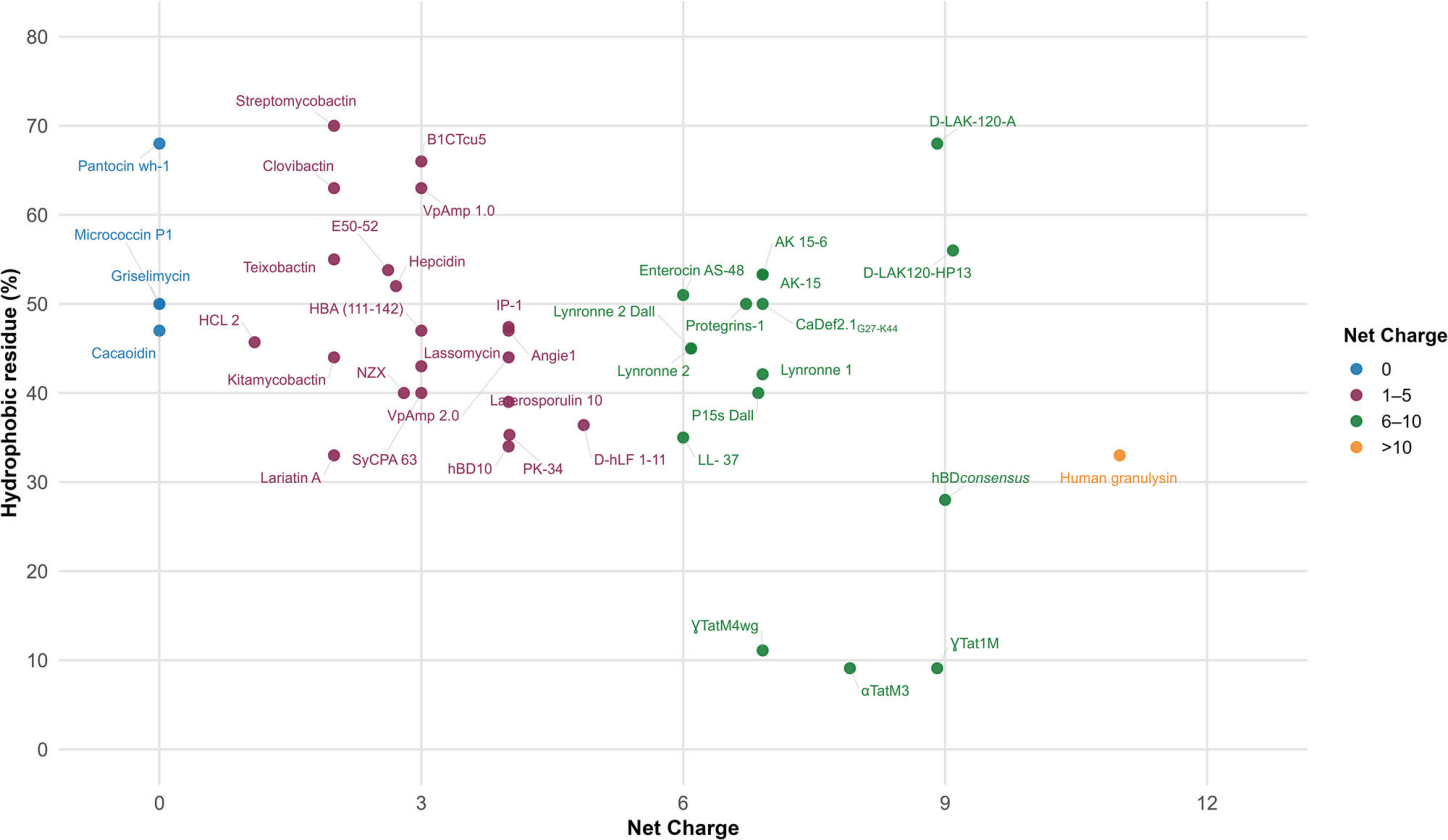
Illustration of peptide charge versus hydrophobicity. A scatter plot showing net charge versus percentage of hydrophobic residues for anti-TB peptides is plotted based on the data from Tables 1 and 2. Peptides are colour-coded by net charge: neutral (0, blue), +1 to +5 (magenta), +6 to +10 (green), and > +10 (orange). This analysis is descriptive and exploratory, intended to visualise broad physicochemical diversity across structurally unrelated peptide templates. Trends observed here should not be interpreted as optimisation rules, as charge-hydrophobicity relationships are highly dependent on primary sequence context and scaffold architecture. Importantly, similar physicochemical properties across distantly related peptides do not imply shared mechanisms of action. This image was created using the ggplot2 package in R-version 4.5.2^194,195^.

A comparison of net charge and hydrophobicity indicates that many anti-TB peptides cluster within a moderate net-charge range (+1 to +9) (Fig. 5) rather than at extremes of cationicity or hydrophobicity. Several active peptides, including Laterosporulin10^103^, B1CTCu5^46^, and Enterocin AS-48^82^, are enriched in Lys (K) alongside hydrophobic residues such as Ala (A) and Leu (L), highlighting common compositional features among subsets of peptides. However, these trends are descriptive and context-dependent, and amino-acid composition alone cannot be used to derive universal design rules across diverse AMP templates.

Within this dataset, 16.66% (7/42) of peptides exhibited hydrophobicity values below 35%, 52.38% (22/42) of peptides displayed hydrophobicity values between 35–50% and 30.95% (13/42) of peptides showed hydrophobicity values >50% (Fig. 5). Based on net charge and hydrophobicity, four groupings can be visualised. Group 1 (blue) comprises neutral peptides with hydrophobicity >50%. For example, neutral peptides Micrococcin-P1, Griselimycin and Pantocin wh-1 exhibited hydrophobicity of 50%, 50%, and 68%, with MICs of 0.063 µM^121^, 6.25 µM^124^, 3.85 µM^125^, respectively. Groups 2 and 3 span a broad hydrophobicity range (10–70%) but are distinguished by net charge (group 2, magenta, +1 to +5; group 3, green, +6 to +10) and together include the majority of peptides analysed. For instance, Lassomycin, with a net charge of +3 and 43% hydrophobicity, displays an MIC of 1.59 µM (0.8–3 µg/mL)^96^.

#### Motif enrichment and sequence patterning

Motif discovery analysis across the complete anti-TB peptide dataset using the STREME algorithm implemented in MEME Suite (5.5.9)^130^ did not identify any conserved or TB-specific sequence motifs. The patterns detected corresponded to generic AMP features, such as cationic residue enrichment or cysteine spacing, rather than determinants uniquely associated with anti-mycobacterial activity. This supports the conclusion that rational optimisation must be performed within defined peptide templates rather than inferred from global motif analysis across structurally diverse sequences.

Overall, based on the available data from APD6 and literature, several trends like composition of AA, percentage of Cys rich residues, motif discovery in the non-phylogenetic peptides, charge hydrophobicity relationship highlights the repetitive highly context-dependent features. While certain compositional trends such as enrichment of cationic residues, moderate hydrophobicity, or conserved Cys motifs within specific peptide families are associated with in vitro antimicrobial activity, no universal sequence or physicochemical signature emerges across structurally diverse AMP templates. These findings reinforce that antimicrobial potency is governed by primary sequence context, residue distribution, and higher-order structural constraints, rather than by individual bulk properties alone. Consequently, the trends identified here should be viewed as descriptive and hypothesis-generating, providing a comparative framework for understanding AMP diversity against Mtb, rather than as transferable design rules or predictors of in vivo efficacy.

## Recommendations and translational framework for overcoming bottlenecks in proposing AMPs as new anti-TB therapeutics

**Fig. 6:**
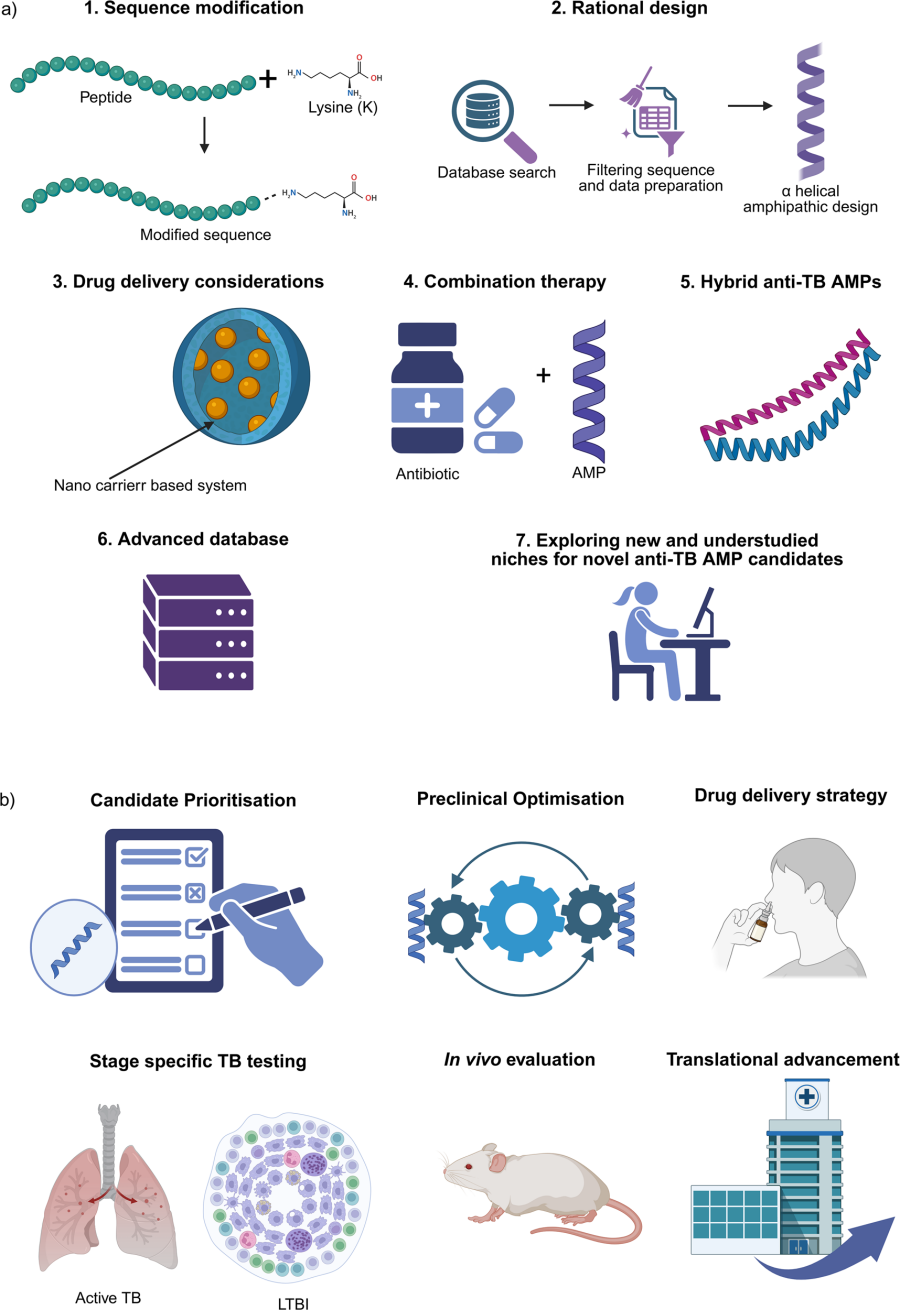
Recommendations and translational framework for advancing anti-TB antimicrobial peptides. **a** Recommendations for design and improvement of anti-TB AMPs. Proposed strategies for overcoming the bottlenecks in proposing AMP as new anti-TB regimen including sequence modification, rational design, combination therapy, synthesising of hybrid anti-TB AMPs, development of a comprehensive database and exploring understudied niches for novel anti-TB candidates. **b** Proposed translational framework for advancing anti TB antimicrobial peptides from in vitro activity to therapeutic development. The framework outlines a staged pathway beginning with candidate prioritisation based on potent anti-TB activity, intracellular efficacy, acceptable selectivity and toxicity, and synergy potential. Subsequent stages include preclinical optimisation, delivery strategy development, disease context specific testing for active TB and LTBI, in vivo evaluation, and translational advancement towards adjunctive use, dose reduction, regimen shortening, and clinical development. This image was created using BioRender.

Although AMPs have significant potential as anti-TB therapeutics, a major challenge in TB drug discovery remains the limited availability of in vivo validation studies. Given the time and complexity required to translate optimised peptides into in vivo evaluation, candidates that already demonstrate favourable in vitro activity, such as those highlighted in this review, can serve as a rational starting point for early-stage optimisation. Accordingly, we outline several proposed strategies (Fig. 6a), including sequence modification and combinatorial formulations, that may inform initial optimisation efforts rather than directly addressing in vivo limitations.

### Recommendations for advancing promising anti-TB AMPs

#### Sequence modification: progress, design lessons and limitations

Only a few anti-TB peptides have undergone sequence modification and optimisation. Most of the peptides reported in Tables 1 and 2 are natural products or minimally altered derivatives. The majority of the reported optimisation strategies primarily aim to improve in vitro potency rather than in vivo performance. Peptide AK-15^131^, listed in Table 2, provides a useful example of sequence optimisation in anti-TB peptide development. In this study, the parent peptide AK15 was subjected to a rearrangement of AA by segregating hydrophobic residues on one face of the peptide and hydrophilic residues on the opposite face, without altering other physicochemical parameters. This design strategy is consistent with reports^132–134^ suggesting that cationic, amphipathic α-helical AMPs are critical for effective interaction with Mtb. As a result of this rearrangement, the optimised analogue AK15-6 exhibited enhanced anti-TB activity (18.75 μg/mL) compared to the parent peptide AK15 (37.5 μg/mL). Moreover, AK15-6 showed improved activity for thermal stability, low toxicity, protection in murine infection models and immune responses in bone marrow-derived macrophages and mice^131^. This example highlights how template-based sequence arrangement can improve in vitro anti-microbial potency. Therefore, systematic screening of existing peptide datasets for similar optimisation opportunities may help to identify analogues with enhanced activity relative to their parent sequences. Another example of sequence modification is seen in Tat peptide analogues^135^; αTatM3, ƔTat1M, ƔTatM4wg (Table 2). The introduction of non-natural surrogates of Lys and Arg at selected positions within the Tat peptide improved anti-mycobacterial activity, while simultaneously enhancing protease resistance and extending peptide half-life. This study shows that template-based substitution of natural cationic residues by non-natural surrogates enhances anti-mycobacterial activity by improving protease resistance and peptide half-life, without increasing peptide length or net charge. Finally, Angie1 is a synthetic peptide identified from an endogenous peptide Angiogenin, which showed activity against both extracellular and intracellular Mtb^75^. This suggests that active peptides can also be generated from truncated sequences to produce synthetic peptides.

In contrast, Teixobactin represents a peptide that has undergone extensive optimisation; however, none of its reported analogues has been evaluated against Mtb. Instead, optimisation efforts have focused on Gram-positive pathogens, with potent activity demonstrated against MRSA^113^. This underscores a key limitation in current optimisation strategies, namely the lack of prioritisation of TB-specific evaluation.

#### Rational design of anti-TB AMPs

Designing an AMP is challenging because peptides can form billions of AA combinations, and it is difficult to identify the accurate combinations with the right balance of charge and hydrophobicity that make the peptide biologically active^136^. Many studies have focused on optimising naturally occurring peptides or screening large synthetic libraries to identify potent therapeutic candidates against a specific pathogen^137,138^. However, more recent advances in computational drug discovery have enabled structure-informed rational design strategies that integrate database mining with three-dimensional (3D) molecular modelling. An example is the use of database filtering technology (DFT) to design peptides that can combat methicillin-resistant pathogens^139^. DFT when combined with protein engineering, rational design and three-dimensional (3D) modelling was successfully employed to engineer AMPs (B1, B2, B3 and B4) with activity against Mtb^139^. Pearson et al. constructed a peptide database comprising natural peptides from APD2^140^ and CAMP^141^, as well as natural and synthetic peptides reported in the literature^142–145^ with anti-Mtb activity. This database was analysed to identify key parameters, including average peptide length, the proportion of hydrophobic and hydrophilic residues per sequence length, and the most frequently occurring amino acids within each category^139^. These analyses identified the rational design of peptides with defined lengths and amino-acid compositions. Structural validation of the designed peptides was performed using PepFold, while three-dimensional models were generated using the Molecular Operating Environment (MOE) software. Peptides B1 and B3 adopted linear α-helical conformations, whereas B2 and B4 formed kinked α-helical structures. Experimental evaluation demonstrated in vitro activity against Mtb, with reported MICs of 4 µM^139^. Importantly, this activity was observed exclusively under in vitro conditions, including in macrophages, and no in vivo efficacy data were reported. This study, therefore, serves as a proof-of-concept for template-guided rational peptide design optimisation against hard-to-treat pathogens, including Mtb, rather than evidence of translational readiness. The principal lesson is that computationally guided optimisation within a defined peptide scaffold^146^ can enhance anti-mycobacterial activity when coupled with experimental validation. However, the absence of data on serum stability, intracellular activity, and pharmacodynamics highlights the substantial gap that remains between in vitro potency and therapeutic applicability.

In addition, peptides active against non-Mtb and closely related proxy strains such as *M. smegmatis, M. bovis* BCG should be further evaluated for their activity against Mtb. For instance, RNase-derived peptides such as RNase3 and RNase6 were active against *Mycobacterium aurum* with intracellular MICs between 5 and 10 µM^147^, and RNase 7 showed activity against *Mycobacterium vaccae* with an MIC of 20.0 ± 0.5 µM^72^. Another peptide Lariatin B showed an MIC of 6.25 µg/mL for *M. smegmatis*^71^. Seeing as proxy strains share similar structural and physiological characteristics with Mtb, rational design of peptides active against these strains may reveal design principles that can be applied to develop novel or more potent anti-TB candidates.

Moreover, features identified from anti-TB peptide analysis, such as cationicity, disulphide bridge formation, amphipathic helices, and specific residue motifs, can inform predictive modelling and aid in the selection or engineering of new anti-TB peptide candidates. An analysis of the sequences of these anti-TB peptides suggests that such motifs could serve as templates for machine learning models, in silico docking screens, or synthetic analogue development. Furthermore, by comparing these peptides to non-anti-TB AMPs with similar structural features, it may be possible to identify overlooked candidates for repositioning as anti-TB agents. Moving forward, these design principles could support the development of a structural framework or scoring matrix to prioritise AMP candidates for experimental validation as anti-TB agents. Such efforts could significantly accelerate the discovery of next-generation peptide therapeutics to combat drug-resistant TB.

#### Drug delivery considerations for anti-TB peptides

A major limitation in the clinical translation of peptide therapeutics is their poor oral bioavailability and short half-life, due to rapid proteolytic degradation in the bloodstream and gastrointestinal tract, as well as clearance by the liver and kidneys^148^. Conventional administration routes may therefore limit effective drug delivery of anti-TB peptides in vivo. For TB, delivery strategies must also account for disease-specific barriers, particularly the intracellular localisation of Mtb within macrophages. To reach intracellular bacteria, AMPs must cross host cell barriers, including macrophage and phagosomal membranes, before accessing and permeabilising the mycobacterial cell envelope^54,149^. This is especially relevant for LTBI, where dormant bacteria may persist intracellularly. However, limited data are available on the activity and mode of action of AMPs in LTBI models, and further investigation is required.

Alternative delivery routes are therefore likely to be important for advancing anti-TB peptides. Pulmonary delivery has emerged as a promising route for anti-TB therapeutics and is particularly attractive because it enables direct targeting of the lungs and infected macrophages. In this context, dry powder inhalation systems have been explored for delivering AMPs such as D-LAK peptides^150,151^, with spray-dried formulations of AMPs showing activity against Mtb in in vitro and ex vivo models. However, further work is required to determine whether such formulations can be translated into clinically applicable inhaler or nasal spray systems.

Nanotechnology-based delivery systems using degradable polymers also offer promise by enabling targeted, sustained drug release at sites of infection^152,153^. Several micro- and nanoparticle-based sustained release systems have been investigated for TB drug delivery. Among these, poly (lactic-co-glycolic acid) (PLGA) is a biodegradable and versatile material, approved by the FDA^154^ and widely used in sustained-release drug delivery systems^154–156^. Although direct evidence for PLGA encapsulated anti-TB peptides remains limited, encapsulation of the AMP plectasin in PLGA enhanced activity against *Staphylococcus aureus*^157^, suggesting that similar approaches may be applicable to anti-TB peptides. Indeed, PLGA microspheres have also been used to deliver rapamycin, alone or in combination with isoniazid, INZ, and rifabutin^158^, while chitosan-based nanocapsules have been developed for BDQ delivery^158–160^. Additional studies have explored PLGA-based delivery of conventional anti-TB drugs, including RPT^161^, RIF^162^ INZ^163,164^, and Moxifloxacin^163^, supporting the wider potential of nanocarrier-based systems for improving anti-TB AMP delivery.

While current evidence for anti-TB AMPs remains largely limited to in vitro and early in vivo studies, advances in pulmonary delivery, nanocarrier systems, and peptide optimisation could help overcome key clinical translational barriers. Future studies should prioritise delivery platforms that improve peptide stability, enhance intracellular targeting, and support effective concentrations at the site of Mtb infection.

#### Combination therapy

Synergy, or combination therapy, involves using two or more active compounds together to achieve a superior effect, aiming to eradicate highly resistant pathogens more effectively^165^. For instance, the D-LAK peptides in combination with INH showed enhanced anti-TB activity against drug-resistant clinical strains of Mtb both in vitro and ex vivo^166^. Furthermore, a recent study reported that a dry powder formulation combining D-LAK peptides with INZ exhibited synergistic antimicrobial activity against multidrug-resistant Mtb clinical isolates, with FICI values ranging from 0.25 to 0.38, demonstrating preclinical efficacy against MDR TB strains^150^. Another anti-TB peptide, laterosporulin10, has synergistic activity with the conventional antibiotic RIF^103^. Notwithstanding, there is limited information available about the combinatorial potential of the anti-TB AMPs reported in the literature, whether with other peptides or conventional anti-TB therapies. There is a need for in-depth studies on the synergistic activities between AMPs with cell permeablilising properties and antibiotics with intercellular targets (BDQ, INZ, RPT) and anti-TB AMPs with cell membrane disruption properties and anti-TB AMPs with intercellular targets (Lassomycin, Micrococcin P1, Griselimycin). Such combinatorial studies may help optimise in vitro screening and identify promising combinations for subsequent in vivo evaluation, thereby advancing the selection of candidate therapeutics for TB.

#### Hybrid AMPs

Another strategy to enhance the activity of AMPs is the rational design of hybrid peptides, which are synthesised by combining different AMPs. For example, a hybrid AMP based on cecropin A, LL-37 and magainin II (CaLL, CaMA, LLaMA and MALL) revealed a broader spectrum antimicrobial activity against an array of species (*Bacillus anthracis*, *Burkholderia cepacia*, *Francisella tularensis* LVS and *Yersinia pseudotuberculosis*) than the parent peptides^167^. Another hybrid AMP synthesised from cecropin A and magainin 2 (CA-MA)^168^and its truncated derivatives was identified to have antibiotic activity and notably affects the expression of efflux pump genes in MDR-TB strain, although further investigations are required to confirm the anti-TB therapeutic potential of CA-MA and its truncated derivatives^169^. Interestingly, the aforementioned hybrid peptides revealed limited haemolysis and cytotoxicity^167–169^. These findings may be leveraged to synthesise hybrid AMPs using a combination of AMPs with shorter sequences or using shorter AMPs and truncated AMPs from long peptides reported for anti-TB activity. This method could present a novel drug design strategy that can be applied for formulating antibiotic-free anti-TB regimens with improved activity against Mtb and drug-resistant variants.

#### Developing a dedicated anti-TB AMP database

Many antimicrobial peptide databases which report on anti-TB AMPs exist, including databases such as APD6 database^55^, DRAMP (Data repository of antimicrobial peptides)^170^, CAMP (Collection of Antimicrobial peptides)^171^, Database of Antimicrobial Activity and Structure of Peptides (DBAASP)^172^. However, there are limitations to each of these databases, such as reporting peptides not experimentally validated as anti-TB peptides or reporting anti-mycobacterial peptides as anti-TB peptides. AntiTbPdb^173^, a database meant to be specific for anti-TB AMPs, for instance, is inaccessible as it is no longer actively maintained. Therefore, the development of an actively maintained, TB-specific database would be valuable. Such a resource could integrate the strengths of existing databases while allowing authors to deposit newly identified peptides, with post-publication validation. This would improve accessibility, support rational peptide design, and ultimately accelerate the development of effective anti-TB therapeutics.

#### Exploring new and understudied niches for novel anti-TB AMP candidates

Uncultured soil bacteria, *Streptomyces* species and human sources have been found to be a great source for anti-TB peptides. For instance, teixobactin^111^ and clovibactin^80^ were obtained from *Eleftheria terrae* ssp, Cacaoidin^116^, Streptomycobactin^95^, Griselimycin^122^, from *Streptomyces* spp. Whereas, Human granulysin^83^, HBA (111–142)^92^, LL-37^104^, D-hLF 1–11^174^, HCL 2^175^ and Hepcidin were obtained from *Homo sapiens*. *Streptomyces* species are recognised as a major repository of structurally diverse natural products^176,177^ due to their robust secondary metabolism^178^. The genus *Actinobacteria* of *Streptomyces* is known to produce two-thirds of the clinically used antibiotics, including RIF and streptomycin^179^. Since *Streptomyces* species is a great reservoir of bioactive compounds, further investigation using artificial intelligence (AI) or machine learning approaches might speed up drug discovery, especially for hard-to-treat pathogens like Mtb.

In addition to soil bacteria, *Streptomyces* species and human sources, other underexplored environments like the rumen microbiome have been identified to have novel AMPs active against multidrug-resistant pathogens^180^. Indeed, a study by Boidin-Wichlacz et al.^181^ reported that D-forms of rumen microbiome-derived peptides showed improved activity against Mtb^181^. Lynronne 2D_*all*_ and P15sD_*all*_ were the most active peptides (Table 2). Similarly, archaeal sources have been reported as an understudied reservoir for novel antimicrobials. A recent study employed APEX 1.1, a deep learning algorithm to identify peptides, including archaeasin-73, with in vivo activity against *Acinetobacter baumannii* loads in mouse infection models^182^. These studies highlight the value of niche ecosystems and the use of AI and deep learning to identify novel peptide sequences capable of targeting Mtb and its drug-resistant variants.

### Bridging promising anti-TB AMPs through a translational framework

The recommendations outlined above highlight that anti-TB AMP development cannot rely on antimicrobial potency alone. To translate promising activity into therapeutic potential, candidate peptides need to be assessed through an integrated framework that considers biological activity, safety, delivery feasibility, disease context, and evidence of in vivo efficacy (Fig. 6b). This is particularly important for TB, where intracellular infection, drug resistance, prolonged treatment regimens, and differences between active disease and LTBI create challenges that are not captured by standard extracellular MIC assays.

Rather than ranking individual peptides, the proposed framework provides a practical structure for organising translational evidence. Peptides with favourable in vitro activity should be prioritised only when this is supported by additional indicators of developability, including intracellular activity, acceptable toxicity, compatibility with formulation strategies, and potential use alongside existing anti-TB drugs. Subsequent evaluation should then determine whether these candidates retain activity in relevant cellular and animal models, achieve appropriate exposure at the site of infection, and demonstrate safety and efficacy under conditions that reflect their intended therapeutic use. In this way, the framework links the optimisation strategies discussed above with the preclinical evidence needed to move anti-TB AMPs beyond promising candidates towards realistic therapeutic development.

## Conclusion

Tuberculosis remains a major global health challenge due to its chronic progression, complex host-pathogen interactions, and increasing levels of AMR. The urgent need for new anti-TB therapeutics is intensified by the limited protection afforded by the BCG vaccine and the declining effectiveness of current drug regimens. Ideally, next-generation anti-TB agents should demonstrate improved efficacy, minimal host toxicity, and a low propensity towards resistance development. In this context, AMPs have emerged as potential alternatives and/or adjuvants for TB treatments.

Although a growing number of anti-TB peptides have been reported to date, only a small fraction have been investigated beyond preliminary in vitro activity assays, and even fewer have progressed to in vivo evaluation. In many cases, antimicrobial activity is defined primarily by MIC values, with limited follow-up on mechanism of action, cytotoxicity, serum stability, or systematic analogue optimisation. Although not peculiar to anti-TB AMPs alone, the use of a standard unit for reporting MIC values (either µg/mL or µM) would greatly facilitate comparison across studies and improve downstream analysis. Moreover, the absence of standardised toxicity data and structured optimisation pipelines hinders the prioritisation of suitable candidates for in vivo studies and represents a major bottleneck in TB peptide research. Furthermore, peptides that display promising in vitro or early in vivo activity may fail in animal models because of poor pharmacokinetics, instability, or host toxicity, limitations that often become apparent only at later stages of development. These challenges highlight the need to integrate considerations of peptide stability, host compatibility, disease-specific contexts (active TB vs LTBI) and delivery strategies early in the discovery process rather than treating them as downstream barriers.

The analyses presented in this review outline practical approaches to improve early-stage screening and candidate prioritisation. Examination of reported anti-TB AMPs reveals recurring descriptive features, including cationicity, enrichment of residues such as Ala, Leu, Lys, and Arg, Cys-rich peptides, and distinct charge-hydrophobicity relationships. Importantly, these features should be interpreted as comparative and context-dependent trends rather than universal design rules, as antimicrobial activity and translational potential remain governed by primary sequence context and peptide-specific properties. In this regard, short synthetic peptides such as AK15 and its optimised analogue AK15-6 provide illustrative examples of template-specific optimisation, showing enhanced anti-mycobacterial activity and reported in vivo efficacy. However, their limited serum stability further underscores the need for continued optimisation to improve pharmacokinetic performance.

Combination and hybrid peptide strategies, including D-LAK peptides, laterosporulin10-based constructs, and cecropin-magainin hybrids, further demonstrate the potential of scaffold-guided approaches to enhance antimicrobial efficacy or achieve synergistic effects. At the same time, there is a clear need for improved and actively curated databases that prioritise validated anti-TB peptides and incorporate mechanistic and toxicity data, distinguishing preliminary activity from more advanced characterisation. Together, these measures would support rational peptide design and accelerate the development of effective anti-TB therapeutics.

Expanding discovery efforts into underexplored ecological niches-such as uncultured soil microorganisms, *Streptomyces* species, and complex microbial ecosystems including the rumen microbiome and archaeome-offers additional opportunities to identify novel peptide scaffolds. Emerging AI and deep-learning approaches may aid in prioritising candidates from these environments, provided that they are guided by high-quality, context-aware datasets rather than MIC-centric annotations alone.

Future research should therefore focus on integrating rational, template-specific optimisation strategies with improved mechanistic understanding, host-peptide interaction studies, and delivery approaches capable of targeting intracellular Mtb without inducing host toxicity. Furthermore, intensified funding and research into in vivo studies are key to advancing anti-TB AMP translation. By aligning peptide discovery with biological constraints and translational requirements, AMPs may be more effectively developed as next-generation therapeutics contributing to the goals of the WHO End TB Strategy.

## Data Availability

All data generated or analysed during this study are included in this article
